# A study on spinal level, length, and branch type of the inferior mesenteric artery and the position relationship between the inferior mesenteric artery, left colic artery, and inferior mesenteric vein

**DOI:** 10.1186/s12880-022-00764-y

**Published:** 2022-03-08

**Authors:** Jie Zhou, Jinghao Chen, Meirong Wang, Feixiang Chen, Kun Zhang, Ruochen Cong, Xiaole Fan, Jushun Yang, Bosheng He

**Affiliations:** 1 Department of Radiology, Affiliated Hospital 2 of Nantong University, No. 6 Hai Er Xiang North Road, Nantong, 226001 Jiangsu China; 2 Department of Radiology, Changzhou Hospital of Traditional Chinese Medicine, Jiangsu, 213000 China; 3 Department of Electrical Engineering, Nantong University, Jiangsu, 226001 China; 4 Clinical Medicine Research Center, Affiliated Hospital 2 of Nantong University, Jiangsu, 226001 China; 5 Nantong Key Laboratory of Intelligent Medicine Innovation and Transformation, Jiangsu, 226001 China

**Keywords:** Dual-energy computed tomography, Monoenergetic plus, Inferior mesenteric artery, Computed tomography angiography

## Abstract

**Background:**

This study was aimed to explore the clinical application of dual-energy computed tomography (DECT) monoenergetic plus (mono+) imaging to evaluate anatomical variations in the inferior mesenteric artery (IMA).

**Methods:**

The clinical and imaging data of 212 patients who had undergone total abdominal DECT were retrospectively analyzed. The post-processing mono+ technique was used to obtain 40-keV single-level images in the arterial phase. Three-dimensional reconstruction was performed to evaluate the relationship between the IMA root position and the spinal level, IMA length, and IMA branch type, as well as the position of the left colic artery (LCA) and inferior mesenteric vein (IMV) at the IMA root level.

**Results:**

The IMA root was located at the L3 level in 78.3% of cases and at the L2/L3 level in 3.3%. The highest vertebral level of IMA origin was L2 (4.2%), and the lowest was L4 (7.1%). The distance from the IMA root to the level of the sacral promontory was 99.58 ± 13.07 mm, which increased with the elevation of the IMA root at the spinal level. Of the patients, 53.8% demonstrated Type I IMA, 23.1% Type II, 20.7% Type III, and 2.4% Type IV. The length of the IMA varied from 13.6 to 66.0 mm. 77.3% of the IMAs belonged to Type A, the adjacent type, and 22.7% to Type B, the distant type.

**Conclusion:**

DECT mono+ can preoperatively evaluate the anatomical characteristics of the IMA and the positional relationship between the LCA and IMV at the IMA root level, which would help clinicians plan individualized surgery for patients.

## Highlights

- DECT mono+ optimal energy level can preoperatively determine the position of the IMA root.
- DECT mono+ can preoperatively evaluate the anatomical characteristics of the IMA.
- DECT mono+ can preoperatively determine the positional relationship between the LCA and IMV at the IMA root level.

## Background

Laparoscopic total mesorectal excision has become the standard therapy for left-sided colorectal cancer and rectal cancer worldwide [1]. Standard operative procedures include resection of the tumor, wide resection of the colonic mesentery, and ligation of the inferior mesenteric vessels [2]. Inferior mesenteric artery (IMA) originates from the front of the abdominal aorta, near its left margin just below the third part of the duodenum at the level of 3rd lumbar vertebra [3]. The management of the IMA is key during the operation, affecting the survival period and postoperative quality of life of patients [4]. Therefore, accurate location of the IMA root and preoperative knowledge of the arterial branch and individual anatomical variations can help surgeons formulate preoperative strategies and perform safe and rapid vessel ligation and lymph node dissection [5, 6].

Currently, there are two IMA ligation options: (1) high ligation with ligation of the left colic artery (LCA), and (2) low ligation, with preservation of the LCA [7]. Low ligation is recommended to protect the blood supply to the anastomosis. Additionally, the incidence of postoperative complications, such as anastomotic fistula, urinary and fecal disorders, and abnormal reproductive function, in patients undergoing low IMA ligation is lower than in those undergoing high ligation [1]. However, low ligation increases the surgical difficulty under laparoscopy because of the lympho-adipose tissue surrounding the IMA trunk and the highly variant pattern of IMA bifurcation, in particular when dense fibrous tissues surround the IMA [8]. Thus, visualization of the length of the IMA trunk and vessel variations is an important concern when performing low ligation. However, the positional relationship between the LCA and inferior mesenteric vein (IMV) varies among individuals [9], either LCA closed to the IMV or LCA distantly lateral to the IMV. Therefore, preoperative understanding of the branching of the LCA and IMV and individual variations in the IMA would be very helpful during surgery. The variations in IMA branching have historically been studied by means of autopsy [10, 11], which cannot be performed in a living patient.

Traditional angiographic examination is an invasive method that cannot provide images of the positional relationship among veins, arteries, and other organs [12, 13]. Owing to the beam hardening effect, traditional computed tomography (CT) angiography results in heavy artifacts and a low contrast between tissues, and thus, cannot easily display clearly the anatomical structure of small supply vessels and the surrounding tissues [14]. Dual-energy (DE) CT monoenergetic (mono) imaging technology can generate virtual monoenergetic images of between 40 and 190 keV, which can substantially improve iodine attenuation at lower kiloelectron volt levels and reduce beam-hardening artifacts at higher kiloelectron volt levels. Additionally, virtual monoenergetic imaging improves contrast attenuation at lower kiloelectron volt levels, which enables better delineation of various vascular structures [15, 16]. However, the low-energy images produced by this technique have a high noise level and a low noise contrast, and therefore, they cannot be widely used in clinical practice. DECT monoenergetic plus (mono+) imaging can improve the contrast of low-energy images and optimize image noise [15–18].

Thus, in this study, we aimed to analyze the anatomical characteristics of the IMA and its positional relationship with the IMV using the DECT mono+ technique to provide preoperative guidance for patients having lesions of the left-sided colon and rectum.

## Methods

### Participant selection

The clinical and imaging data of 227 patients who underwent total abdominal dual-energy enhanced CT examination at the Second Affiliated Hospital of Nantong University from November 2019 to June 2021 were retrospectively collected. The study was approved by the Ethics Committee of the Second Affiliated Hospital of Nantong University (approval number: 2020YKS024), and written informed consent was obtained from all patients. All methods were carried out in accordance with relevant guidelines and regulations.

### Inclusion and exclusion criteria

The inclusion criteria were as follows. (1) Patients with complete general linear data. (2) Patients underwent dual-energy computed tomography angiography (CTA) and their data included complete post-processing images. (3) Patients were older than 18 years. Patients demonstrating (1) previous abdominal surgery, (2) severe abdominal disease that affects image quality, (3) spinal disease and lumbar disc surgery history, or (4) allergy to iodine contrast agent were excluded.

### Preparation before CT examination

The patients were instructed to fast for 4–8 h before the examination. Before the preparatory procedure of intestinal filling, the presence of contraindications for intestinal filling, such as intestinal obstruction and poor tolerance, was determined. To those patients showing no contraindications, 2000 mL of 2.5% isosmotic mannitol was administered orally 1 h before examination (500 mL at 15 m intervals).

### Dual-energy CT scanning protocol

Dual-energy CTA was performed using a dual-source CT system (Somatom Force, Siemens Healthcare, Forchheim, Germany). The scanned area was from the diaphragmatic dome to the lower margin of the pubic bone. The patients were maintained in the supine position. The potentials of bulb tubes A and B were 90 kV and Sn150 kV, respectively, and the currents of bulb tubes A and B were 144 mA and 90 mA, respectively. The linear fusion coefficient was 0.5, the pitch was 1.0, and the gantry rotation time was 0.5 s. All scans were performed with a detector collimation of 2 × 192 × 0.6 mm. The acquisition layer thickness and acquisition space were both 1 mm, and the monitoring technology used was three-phase enhanced scanning. 1.5 mL/kg iopromide (370 mgI/mL) was injected at a rate of 3.5 mL/s, followed by 20–30 mL normal saline.

### Image post-processing

After scanning, the arterial phase data of the IMA-enhanced scanning were transferred to a Siemens Synovia post-processing workstation. The 40-keV level images were selected by post-processing mono+ technology for 3D reconstruction, and multiplanar recombination (MPR) cross-sectional and sagittal images with a layer thickness of 1 mm and layer spacing of 1 mm were obtained. Additionally, IMA curve planar reformation (CPR) images, oblique coronal thin slab maximum intensity projection (thin-MIP) images (parallel to the long axis of the IMA; layer thickness, 10 mm; layer spacing, 5 mm), and volume reconstruction (VR) images of bone removal where the lumbar volume was retained were also obtained.

### Measurement methods

**Fig. 1:**
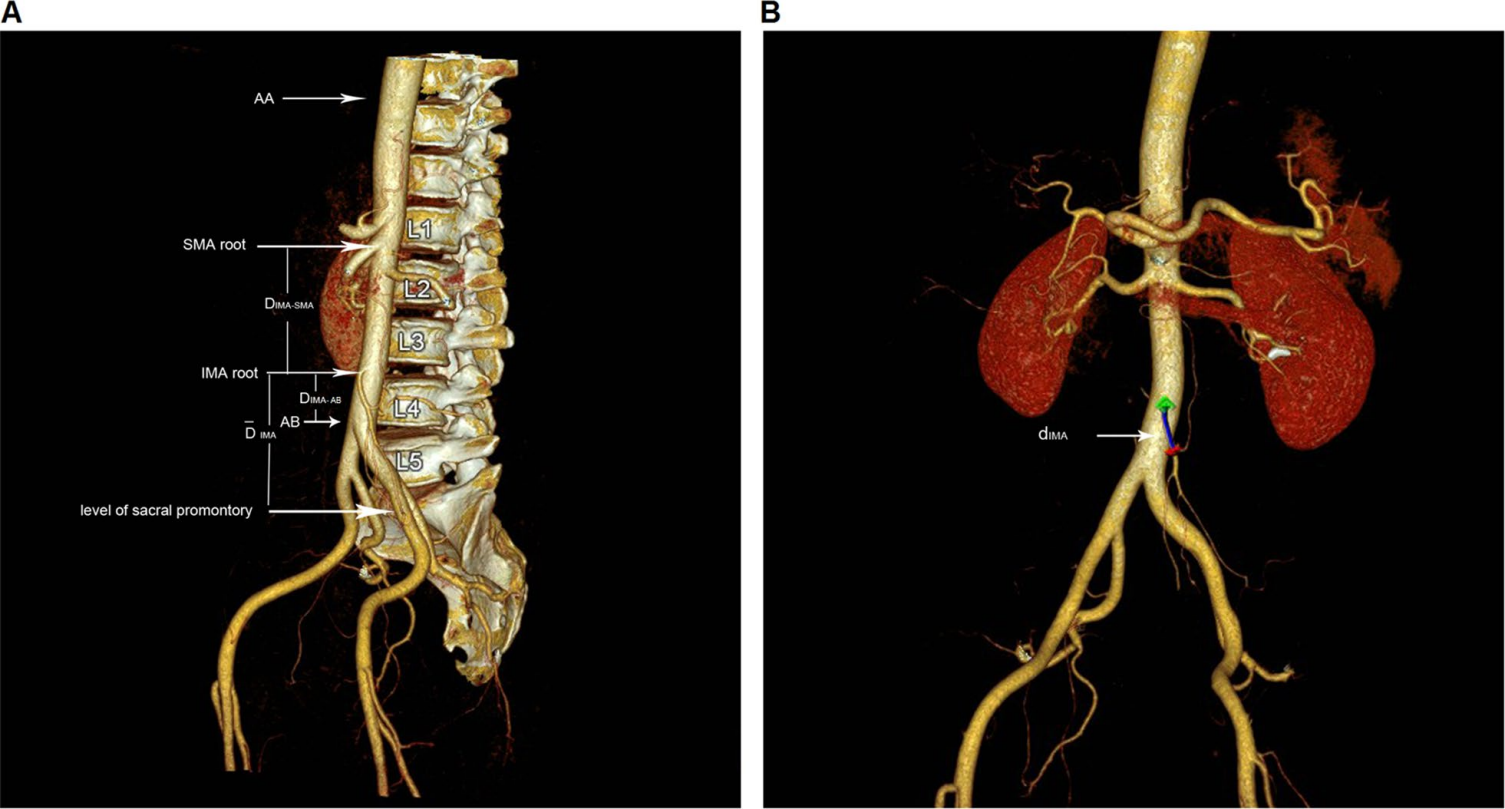
**A** The vertebral level of the inferior mesenteric artery root was determined by the volume reconstruction technique, and the values of D_IMA-SMA_ (the vertical distance from IMA root to SMA root), D_IMA-AB_ (the distance from IMA root to the bifurcation of AA), and $\overline{\mathrm{D} }$_IMA_ (the distance from IMA root to the level of the sacral promontory) were measured. **B** Distance from the root of the IMA to the first branch of the IMA (d_IMA_). *IMA* inferior mesenteric artery, *SMA* superior mesmesic artery, *AA* abdominal aorta, *AB* the bifurcation of the abdominal aorta

Data measurement on VR and CPR images were performed by a radiologist with 3 years’ abdominal work experience. Each data was measured 3 times and averaged to reduce errors. In the VR images, the vertical distance from the IMA root to the superior mesenteric artery (SMA) root (D_IMA-SMA_), distance from the IMA root to the bifurcation of the abdominal aorta (AB) (D_IMA-AB_), and distance from the IMA root to the level of the sacral promontory ($\overline{\mathrm{D} }$_IMA_) were measured (Fig. 1A). In the CPR images, the distance from the IMA root to the first branch of the IMA (d_IMA_) was measured (Fig. 1B). Additionally, the location of the IMA root, IMA typing, and the positional relationship between the IMA root LCA and IMV were confirmed on VR images by another radiologist with 3 years’ abdominal work experience who was not involved in the data measurement and a gastrointestinal surgeon having 8 years’ abdominal work experience. Disagreement was determined through consultation.

**Fig. 2:**
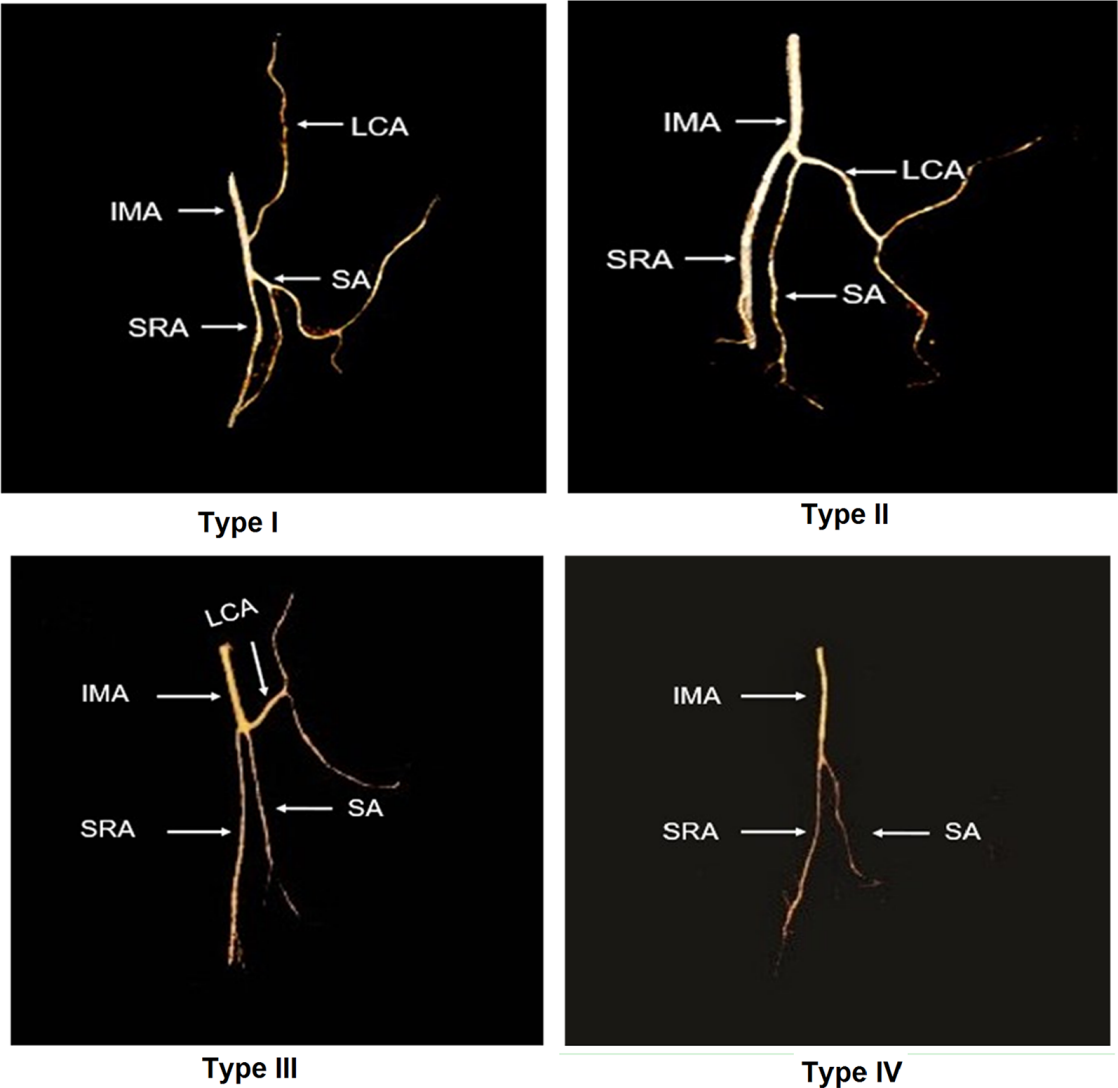
Branch types and distribution of IMA. *IMA* inferior mesenteric artery, *LCA* left colic artery, *SA* sigmoid artery, *SRA* superior rectal artery

**Fig. 3:**
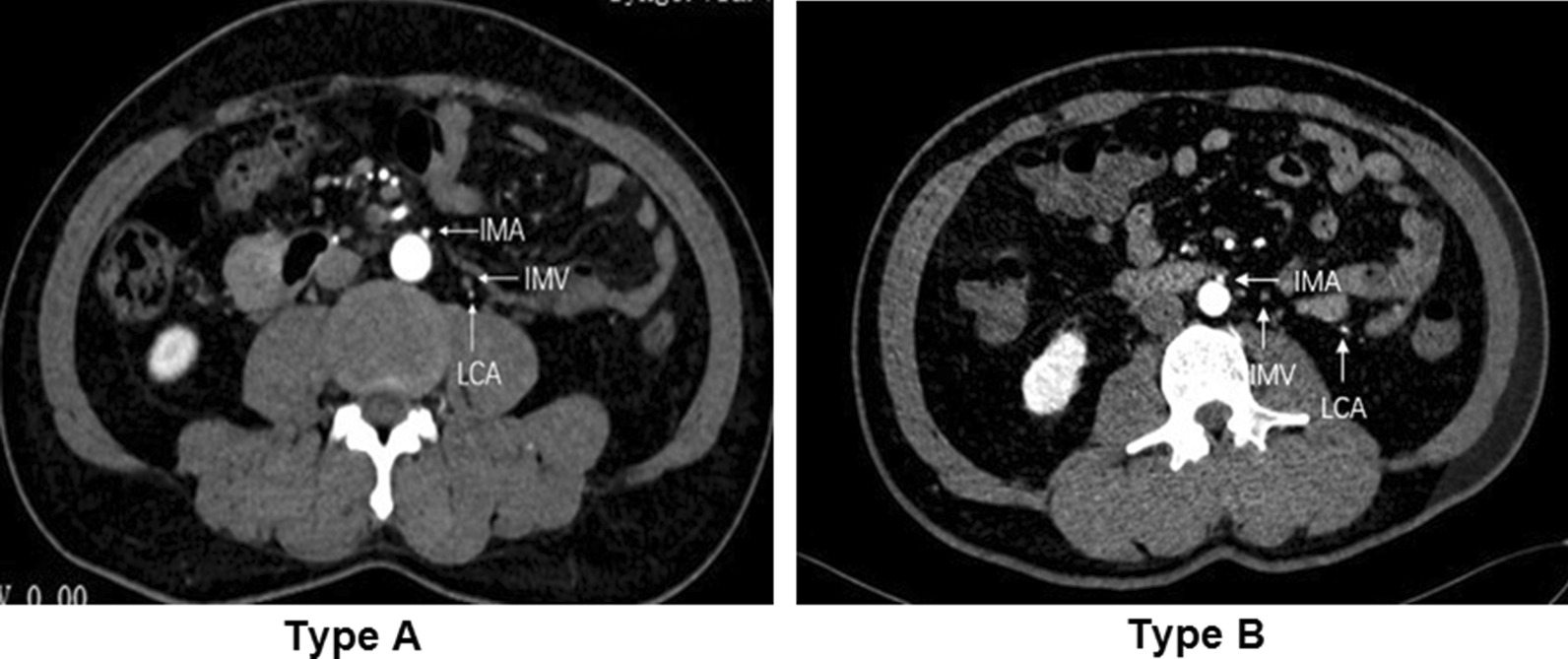
The positional relationship and distribution of the LCA and IMV at the IMA root level. *IMA* inferior mesenteric artery, *IMV* inferior mesenteric vein, *LCA* left colic artery

IMA typing was performed on the thin-MIP images. The IMA branch types were determined according to the classical four types [19]: Type I (issuing from the LCA alone), Type II (issuing from the LCA together with arteriae sigmoideae), Type III (issuing from the LCA together with the arteriae sigmoideae and superior rectal artery), and Type IV (having no LCA) (Fig. 2). The positional relationship between the IMA root LCA and IMV was analyzed on the MPR axial image. The positional relationship between the LCA and IMV can be divided into two types, as shown in Fig. 3: Type A, where the LCA is close to the IMV (distance < 15 mm) and Type B, where the LCA is distantly lateral to the IMV (distance > 15 mm) [20].

### Statistical analysis

Statistical analyses were performed using SPSS 25.0. The measurement data conforming to the normal distribution are presented as mean ± standard deviation. An independent sample *t* test was used to compare two groups, one-way analysis of variance was used to compare multiple groups, and Tukey’s method was used for pair comparison of multiple groups. Categorical variables are presented as the number of cases (percentage). The chi-square test was applied to evaluate the differences between two groups. Multivariate logistic regression was used to analyze the correlation between D_IMA-AB_, d_IMA_, and D_IMA-SMA_ and the IMA root level LCA and IMV position. Differences were considered statistically significant at *p* < 0.05.

## Results

### Clinical data

A total of 15 patients did not meet the criteria owing to various factors: 8 patients had severe spinal disease, 4 patients had previously undergone abdominal surgery, and 3 patients suffered severe abdominal disease affecting image quality. These patients were excluded. Finally, 212 patients were enrolled in the study, including 116 males (aged 21–85 years; average age 59.22 ± 12.77 years) and 96 females (aged 19–88 years; average age 59.31 ± 14.32 years) (Table 1).

**Table 1:** General clinical data of 212 patients

Parameter	Male (n = 116)	Female (n = 96)
Age, mean (years)	59.22 ± 12.77	59.31 ± 14.32
Height, mean ± SD (cm)	170.37 ± 5.92	159.97 ± 5.59
Body mass, mean ± SD (kg)	73.09 ± 10.69	61.73 ± 13.36
Body mass index, mean ± SD (kg/m^2^)	25.13 ± 3.1	24.11 ± 4.92
Normal total abdominal	31	25
Hepatic cyst	33	29
Cavernous angioma	2	1
Cholecystitis combined with gallstones	2	2
Gallstone	11	6
Suprarenoma	2	3
Kidney stone	13	14
Renal cyst	15	9
Ureteral calculus	7	4
Myoma of uterus	0	3

### Relationship between IMA root position and vertebral level

Among the 212 patient cases described above, 166 cases in which the IMA originated from the abdominal aorta at the L3 vertebral body level accounted for the highest proportion (78.3%). Seven cases, where the IMA originated from the abdominal aorta at the L2/L3 disc level accounted for the lowest proportion (3.3%). The highest vertebral level of IMA origin was the L2 vertebral body, and the lowest vertebral level was the L4 vertebral body, as shown in Table 2.

**Table 2:** Relationship between IMA roots at different vertebral levels and their distance to aortic bifurcation, sacral promontory, SMA roots and the first branch of IMA (mm)

Distance to IMA root	L2 vertebral body (n = 9)	L2/L3 disc level (n = 7)	L3 vertebral body (n = 166)	L3/L4 disc level (n = 15)	L4 vertebral body (n = 15)	*F*	*P*
D_IMA-AB_, mean ± SD	42.55 ± 4.01	42.62 ± 11.18	41.95 ± 6.76	41.67 ± 5.76	36.71 ± 4.42	2.22	0.068
D_IMA,_ mean ± SD	117.18 ± 9.19	116.34 ± 6.63	100.87 ± 10.86	87.02 ± 5.38	79.39 ± 11.06	31.01	0.000
D_IMA-SMA_, mean ± SD	67.76 ± 11.11	68.14 ± 11.71	72.04 ± 8.89	76.25 ± 8.76	85.12 ± 9.31	8.82	0.000
d_IMA_, mean ± SD	46.03 ± 11.36	42.37 ± 7.72	38.73 ± 10.03	36.52 ± 7.51	31.51 ± 7.91	3.72	0.006

The relationship between the IMA root position and the vertebral level was analyzed, including D_IMA-AB_ (41.60 ± 6.73 mm), $\overline{\mathrm{D} }$_IMA_ (99.58 ± 13.07 mm), d_IMA_ (38.49 ± 9.99 mm), and D_IMA-SMA_ (72.96 ± 9.75 mm). $\overline{\mathrm{D} }$_IMA_, d_IMA_, and D_IMA-SMA_ at different vertebral levels showed statistically significant differences (*P* < 0.001, *P* < 0.001, and *P* = 0.006, respectively). $\overline{\mathrm{D} }$_IMA_ increased with an increase in the vertebral level and L2 vertebral body had the longest $\overline{\mathrm{D} }$_IMA_. The pairwise comparison of $\overline{\mathrm{D} }$_IMA_ at different vertebral levels showed a statistically significant difference (*P* < 0.001). No statistical significance was found in the pairwise comparison of d_IMA_ and D_IMA-SMA_ at different vertebral levels. The values of D_IMA-AB_, $\overline{\mathrm{D} }$_IMA_, d_IMA_, and D_IMA-SMA_ at the various vertebral levels are shown in Table 2. L4 vertebral body had the longest D_IMA-SMA_ and L2 vertebral body had the longest d_IMA_.

### Relationship between IMA typing and IMA length

One hundred and fourteen patients (53.8%, the highest proportion) demonstrated Type I IMA, 5 (2.4%, the lowest proportion) demonstrated Type IV IMA, 49 (23.1%) demonstrated Type II IMA, and 44 (20.7%) demonstrated Type III IMA. The relationship between IMA typing and d_IMA_ was analyzed. The d_IMA_ value of Type I IMA was 35.59 ± 8.99 mm, of Type II IMA was 42.59 ± 10.06 mm, of Type III IMA was 40.96 ± 10.36 mm, and of Type IV IMA was 42.84 ± 8.96 mm. A significant difference was found among the four types of d_IMA_ (*P* < 0.001), and Type I IMA had the shortest d_IMA_. Pairwise comparison showed that the d_IMA_ value of Type I IMA was less than that of the other three types (*P* < 0.05). No significant difference in d_IMA_ among Types II, III, and IV was shown. Moreover, Types II, III, and IV were further combined, with a length of 41.87 ± 10.08 mm. Type I d_IMA_ was significantly shorter than non-Type I (*P* < 0.001).

### Relationship between LCA and IMV positional relationship at IMA root level and clinical features

Of the 212 patients, 5 cases without LCA were excluded, and the data of the remaining 207 were analyzed for the positional relationship between the LCA and IMV in the IMA root. There were 160 cases (77.3%) of Type A IMA, the adjacent type and 47 cases (22.7%) of Type B, the distant type. We further evaluated the relationship between the LCA and IMV positional relationship at the IMA root level and the clinical characteristics of the subjects. As shown in Table 3, the IMA that the LCA was distantly lateral to the IMV at the root level was significantly correlated with the IMAs that had a longer D_IMA-AB_ and d_IMA_ and shorter D_IMA-SMA_. The different positions of the LCA and IMV were not related to gender, age, height, body mass, body mass index, and IMA type. Additionally, D_IMA-AB_, d_IMA_, and D_IMA-SMA_ were included to construct the multivariate logistic regression equation. The results showed that d_IMA_ was an independent influencing factor for two different positional relationships of the LCA and IMV at the IMA root level (OR = 1.063, 95% CI 1.024–1.102, *P* = 0.001) (Table 4).

**Table 3:** The relationship between LCA and IMV position relationship at IMA root level and the clinical characteristics of the subjects

	Type A adjacent type (n = 160)	Type B distant type (n = 47)	*t/χ*^*2*^	*P*
Gender			0.395	0.53
Male, n (%)	90 (56.3%)	24 (51.1%)		
Female, n (%)	70 (43.7%)	23 (48.9%)		
Age, mean ± SD (years)	59.38 ± 13.30	59.59 ± 14.12	− 0.093	0.926
Height, mean ± SD (cm)	165.72 ± 8.02	165.23 ± 7.14	0.378	0.706
Body mass, mean ± SD (kg)	67.61 ± 12.37	68.86 ± 16.24	-0.564	0.573
Body mass index, mean ± SD (kg/m^2^)	24.51 ± 3.43	25.19 ± 5.78	− 1.008	0.314
D_IMA-AB_, mean ± SD (mm)	41.06 ± 6.76	43.48 ± 6.56	− 2.162	0.032
D_IMA_, mean ± SD (mm)	99.25 ± 13.29	101.12 ± 13.09	− 0.852	0.395
D_IMA-SMA_, mean ± SD (mm)	73.58 ± 9.84	70.19 ± 9.24	2.107	0.036
d_IMA_, mean ± SD (mm)	36.88 ± 9.93	43.54 ± 8.59	− 4.164	0.000
IMA type			0.398	0.819
Type I	90 (56.3%)	24 (51.1%)		
Type II	37 (23.1%)	12 (25.5%)		
Type III	33 (20.6%)	11 (23.4%)

**Table 4:** The relationship between the multi-factor clinical characteristics and the LCA and IMV position relationship at IMA root level

	b	SE	Wald	OR	95% CI	*P*
D_IMA-AB_	0.034	0.027	1.637	1.035	0.982–1.091	0.201
d_IMA_	0.061	0.019	10.484	1.063	1.024–1.102	0.001
D_IMA-SMA_	− 0.019	0.019	3.455	0.981	0.946–1.018	0.305

### Length of the IMA

The length of the IMA (d_IMA_) varied greatly, with a minimum of 13.6 mm, a maximum of 66.0 mm, and an average of 38.49 ± 9.99 mm. Among 116 males, the d_IMA_ was 18.6–55.6 mm, with an average of 37.45 ± 9.02 mm. In 96 female patients, the d_IMA_ was 13.6–66.0 mm, with an average of 39.77 ± 10.98 mm. No statistically significant difference was found between the two groups (*P* = 0.093).

## Discussion

Laparoscopic surgery for left-sided colorectal cancer and rectal cancer has gained wide clinical acceptance because of its minimal invasiveness, reduced blood loss, and the relatively short hospital stay involved [21]. However, misidentification and misligation of mesenteric vessels are the main reasons for massive abdominal bleeding, prolonged operation time, and poor postoperative prognosis [22]. Therefore, preoperative awareness of the anatomical structure of the IMA and its positional relationship with the IMV preoperatively, which helps surgeons formulate preoperative strategies and perform safe and rapid vessel ligation, is required. This study was based on DECT mono+ technique to evaluate the anatomy of IMA and its surrounding vessels.

During radical resection of left-sided colorectal cancer and rectal cancer, identification of the location of the IMA root is the first step in the management of the IMA. The present study revealed that the IMA origin is between the L2 and L4 intervertebral disc levels, and the majority (78.3%) of IMAs are located at the L3 level, which is consistent with the results of Ekingen et al. [23]. Additionally, previous studies have also measured the mean distances from the IMA root to the SMA root, aorta abdominalis branch, and the level of sacral promontory, which were 70.80 ± 0.90 mm, 42.0 ± 8.5 mm, and 101.8 ± 14.0 mm, respectively [23, 24]. In accordance with the results above, the three mean distances were 72.96 ± 9.75 mm, 41.60 ± 6.73 mm, and 99.58 ± 13.07 mm, respectively. Although the SMA root, aorta abdominalis branch, and the level of sacral promontory can be used as location markers for IMA roots, the SMA root and aorta abdominalis branch are located behind the peritoneum, requiring a deeper dissociative Toldt space [25]. The level of the sacral promontory serves as a marker of the entrance to the pelvis and is more easily determined. Importantly, we found that the distance between the IMA root and the level of the sacral promontory increased with the elevation of the IMA roots at the vertebral level. Therefore, preoperative prediction of the location of the IMA root and its distance from the level of the sacral promontory by the optimal energy level of the DECT mono+ technique can help clinicians locate the IMA quickly.

After the location of the IMA root, the ligation method of the IMA was selected. Currently, two different levels of ligation are commonly used. High ligation is obtained with transection of the IMA 1 cm distal to the aorta, associated with the transection of the IMV at the inferior border of the pancreas. Low ligation is obtained with the transection of the IMA 1 cm distal to the origin of the LCA to allow preservation of the LCA [26]. The branching type of the IMA significantly affects the choice of ligation method for the IMA [6]. In this study, Type I accounted for 53.8% of the IMAs (the majority), followed by Types II and III (23.1% and 20.7%, respectively), and Type IV accounted for only 2.4% (the least), which is consistent with previous reports that the proportion of the four types of IMA is 44.6–55.8% for Type I, 11.5 to − 22.5% for Type II, 28.5 to − 31.3% for Type III, and 1.6–2.4% for Type IV [20, 24, 27]. Additionally, in a study in which IMA branching was studied by dissecting cadavers, the reported frequencies of Types I, III and IV, were 41–56%, 38–50%, and 0–6% [28, 29]. In a recent study of Murono et al. [9], 5.1% of patients lacked the LCA (Type IV) and that the majority of the cases with an LCA were classified as type I or type III (41.2% and 44.7%). These results are basically consistent with the results of the DECT mono+ in the present study. If the IMA is known to be of Type IV (without an LCA) before surgery, high ligation and thorough dissection of the lymph nodes at the root of the IMA can be performed directly to avoid a time-consuming intraoperative search for the LCA and reduce the risk of accidental injury to peripheral blood vessels and nerves [30].

During the low ligation procedure, an exposed IMA is required for dissection of the lymph nodes around it, which is technically demanding and time consuming and affects the postoperative survival of patients [31]. The time required for intraoperative dissection of the IMA peripheral lymph node is related to the length of the exposed IMA (the distance from the IMA root to the first branch of the IMA) [32]; thus, the length of the IMA was the focus of this study. It was found that the IMA length varied greatly among patients, ranging from 13.6 to 66.0 mm (average length 38.49 ± 9.99 mm). In the study of Murono et al. [9], the length of the IMA widely varied from 10.1 to 82.2 mm (median, 38.4 mm). In a cadaveric study, the lengths were reported to range from 3 to 5 cm [11, 13]. We also analyzed the relationship of the IMA length among different IMA types and found that the length of Type I IMA (35.59 ± 8.99 mm) was significantly less than that of the other three types (II: 42.59 ± 10.06 mm; III: 40.96 ± 10.36 mm; and IV: 42.84 ± 8.96 mm). As a result, preoperative evaluation of the IMA type and length could increase the confidence of the surgeon, avoid unnecessary exposure of the IMA, and shorten the operation time.

During laparoscopic radical resection, most gastrointestinal surgeons routinely dissect the LCA from the medial to the lateral part and then ligate it distally. An ambiguous positional relationship between the LCA and IMV easily results in accidental injury to the IMV [33, 34]. Therefore, the relationship between the LCA and IMV is an additional key point in low ligation. In this study, Type A IMAs constituted 77.3% and Type B 22.3% of cases, which is consistent with the results of Murono et al. [9] (71.4% for Type A and 28.6% for Type B). Further study revealed that the IMA length was an independent factor affecting the relationship between the IMV’s position and the LCA at the root level of the IMA (OR = 1.063, 95% CI 1.024–1.102, *P* value = 0.001). In cases where the IMA is longer, the LCA at the root level is usually located distant from the IMV. Preoperative understanding of the positional relationship between the LCA and IMV is conducive to accurate vascular ligation by clinicians.

There were, however, some limitations to this study. On the one hand, the anatomical characteristics of the IMA shown by DECT mono+ optimal energy level were not compared with the actual intraoperative situation to prove the accuracy of the DECT mono+ technique. On the other hand, no further analysis of the specific effects of the anatomical characteristics of the IMA (such as the branching type of IMA) is conducted for the intraoperative and postoperative outcomes of patients with colorectal cancer. Additionally, the sample size in this study was a little small and more patients will be included in the further study. Last, we did not perform comparison with images reconstructed at 70 or 75 keV. Our previous study has compared the signal-to-noise ratio (SNR), contrast to noise ratio (CNR) and subjective scoring among conventional 120 kVp linear fusion images, 40 keV, 50 keV, 60 keV, 70 keV, 80 keV and 90 keV single energy level images. Our results suggested that 40 keV had the highest CNR (*P* < 0.05). Additionally, the subjective score of 40 keV single energy level image was significantly higher than that of the other 6 groups (*P* < 0.05) (Data has not been published). These results further indicated that dual-energy CT mono+ 40 keV single-level image could significantly optimize IMA display.

## Conclusion

In conclusion, DECT mono + optimal energy level can preoperatively determine the position of the IMA root, evaluate the IMA type, measure the IMA length, determine the positional relationship between the LCA and IMV, and help clinicians make individualized surgical plans for patients.

## Data Availability

The data that support the findings of this study are available on request from the corresponding author.

## Abbreviations

DECT: Dual-energy computed tomography
mono+: Monoenergetic plus
IMA: Inferior mesenteric artery
LCA: Left colic artery
IMV: Inferior mesenteric vein
CT: Computed tomography
MPR: Multiplanar recombination
CPR: Curve planar reformation
thin-MIP: Thin slab maximum intensity projection
VR: Volume reconstruction
SMA: Superior mesenteric artery
